# Prospect of stem cells conditioned medium (secretome) in ligament and tendon healing: A systematic review

**DOI:** 10.1002/sctm.19-0388

**Published:** 2020-04-18

**Authors:** Sholahuddin Rhatomy, Thomas Edison Prasetyo, Riky Setyawan, Noha Roshadiansyah Soekarno, FNU Romaniyanto, Andi Priyo Sedjati, Tito Sumarwoto, Dwikora Novembri Utomo, Heri Suroto, Ferdiansyah Mahyudin, Cita Rosita Sigit Prakoeswa

**Affiliations:** ^1^ Department of Orthopaedics and Traumatology Dr. Soeradji Tirtonegoro General Hospital Klaten Indonesia; ^2^ Faculty of Medicine, Public Health, and Nursing Universitas Gadjah Mada Yogyakarta Indonesia; ^3^ Soeradji Tirtonegoro Sport Center and Research Unit Dr. Soeradji Tirtonegoro General Hospital Klaten Indonesia; ^4^ Department of Orthopaedics and Traumatology Dr. Chasbullah Abdulmadjid General Hospital Bekasi Indonesia; ^5^ Department of Orthopaedics and Traumatology Surakarta Indonesia; ^6^ Faculty of Medicine Universitas Sebelas Maret Surakarta Indonesia; ^7^ Department of Orthopaedics and Traumatology Dr. Soetomo General Hospital Surabaya Indonesia; ^8^ Faculty of Medicine Universitas Airlangga Surabaya Indonesia; ^9^ Department of Dermatology and Venereology Dr. Soetomo General Hospital Surabaya Indonesia

**Keywords:** conditioned medium, ligament, secretome, stem cell, tendon

## Abstract

**Background:**

Tendon or ligament tears can decrease patients' quality of life. Many therapeutic interventions are available to treat such injuries. Mesenchymal stem cells (MSCs) have been shown to be effective in treating tendon or ligament tears; however, the use of stem cell‐conditioned medium (CM) requires further investigation. This review focused on the use of stem cell CM as treatment for tendon or ligament tears.

**Methods:**

A systematic literature search was performed on PubMed (MEDLINE), OVID, EMBASE, the Cochrane Library, Scopus, Web of Science, and Science Direct with the terms *conditioned media* or *conditioned medium* or *secretome* or *microvesicle* or *extracellular vesicle* or *exosome*, and *tendon* or *ligament* as the search keywords. A total of 852 articles were reviewed. Five articles were identified as relevant for this systematic review.

**Results:**

Meta‐analysis could not be performed because of the high heterogeneity of the reviewed studies; however, the results of this study support a positive effect of conditioned media in tendon and ligament treatment.

**Conclusion:**

This review provides evidence of improvement in the tendon and ligament healing process with stem cell CM therapy in preclinical studies.

## Graphical abstract

The secretome or stem cells conditioned medium comprises of soluble proteins and secreted extracellular vesicles. The proteins include biologically active factors such as cytokines, chemokines, and growth factors. The vesicular factors include exosomes and microvesicles (Driscoll J, Patel T. The mesenchymal stem cell secretome as an acellular regenerative therapy for liver disease. *J Gastroenterol*. 2019;54(9):763‐773. doi: 10.1007/s00535‐019‐01599‐1).

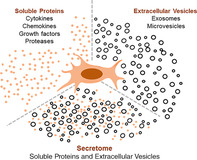

**Significance statement**

Tendon or ligament tear can decrease patients' quality of life. Many therapeutic options are available to treat such injuries. Mesenchymal stem cells (MSCs) have been shown to be effective in treating tendon or ligament tears; however, the use of MSC conditioned medium (CM) needs to be investigated. This review proposes the use of MSC CM as a treatment option for tendon or ligament tear.

## 1 INTRODUCTION

Tendon or ligament tears are common injuries that can diminish patients' quality of life. The healing process after injury may not be adequate, thus leading to the formation of scar tissue. Many therapeutic interventions are available for treating such injuries, including physiotherapy, oral pain medication, steroid injection, and surgical correction; however, some treatments do not provide a satisfactory result. Nonoperative treatments often have an unsatisfactory result and only serve for pain control.^1^, ^2^ Reconstruction surgery may be performed to reattach a tendon; however, the functional outcome may be compromised by tendon and muscle strength deterioration because the tendon was harvested.^1^

Stem cell‐based therapy has shown promise as an adjunct treatment due to its self‐renewal properties, differentiation potentials, and immunomodulatory activities.^3^ A systematic review by Ahmad et al^4^ concluded that stem cell treatment was also applicable for tendon healing in animal and human subjects. Among stem cells, mesenchymal stem cells (MSCs) are particularly useful for tendon and ligament healing.^1^ Extensive studies have shown the therapeutic effect of stem cells in many types of tissue injury; however, the survival ability of MSCs was too short to have an effective and long‐term impact. The main effect of stem cells is probably mediated by the paracrine mechanism, which is known as stem cells conditioned medium (CM) or secretome.^5^

CM or secretome is a medium where the stem cells are cultured and composed of soluble proteins, lipids, nucleic acids, and extracellular vesicles (EV) or microvesicles (MVs).^6^, ^7^ Those vesicles are further categorized into exosomes and shedding vesicles. Meticulous studies on EVs and exosomes have also demonstrated potential regenerative effects, including an anti‐inflammatory effect and tendon healing.^5^, ^8^ There is some clinical benefit of CM (secretome) usage. It could resolve safety considerations associated with the transplantation of stem cells, such as tumorigenicity, transmission of infections, and immune incompatibility.^5^ CM (secretome) could be produced in large amounts and stored for a long period of time without loss of product potency and without toxic cryo‐preservative agents. It may be also modified for desired effects and can reduce cost and time for production and maintenance of cell‐based therapy.^5^

Previous in vitro studies of stem cells CM or secretome usage showed promising outcomes. Studies by Chen et al^9^ and Shimode et al^10^ reported cell rat tenocytes proliferation; both studies showed significant improvement for the bone marrow (BM) MSC CM groups compared with the control groups. These studies found that cell viability was increased significantly in the CM‐treated groups compared with controls. These studies also mentioned significant reduction of inflammatory markers with CM treatment. A study by Wang et al showed significant increase of tissue inhibitor of metalloproteinase (TIMP), an anti‐inflammatory protein, that could improve biomechanical properties.^11^ Tenogenic differentiation was also enhanced significantly in cells treated with CM, as has been shown by a significant increase of Col‐I expression and other tenogenic markers in some studies.^9^, ^10^, ^11^

Studies by Lange‐Consiglio et al^12^ and Shen et al^13^ showed significant reduction in peripheral blood mononuclear cell (PBMC) proliferation in CM treatment compared with control. Interestingly, the study by Lange‐Consiglio et al found that EV did not significantly inhibit PBMC proliferation, compared with control.^12^ The study by Shen et al found that nuclear factor κB activity of macrophages was inhibited significantly by EV, compared with control.^13^

Stem cell therapy is an emerging therapeutic modality, and a systematic review of existing preclinical studies is needed to determine safety and efficacy and, ultimately, to guide future studies. The main purpose of this review was to systematically summarize the best available evidence in vivo studies regarding the use stem cells CM or secretome for the treatment of any tendon or ligament injury. It was hypothesized that the application of stem cell CM would promote tendon and ligament healing in animal models.

## 2 METHODS

### 2.1 Eligibility criteria

The inclusion criteria for this review consisted of the following:

- Study design: controlled animal (in vivo study).
- Study group: animals with any tendon or ligament injuries (by surgery or primary injury).
- Interventions: any application of stem cell‐conditioned media (CM), EVs/MVs, or exosomes to the study groups.
- Outcomes: main outcomes were any functional, biomechanical, and safety outcomes.
- Language: English

Non‐English studies, duplicates, review studies, and irrelevant articles were excluded.

### 2.2 Literature search and study selection

A comprehensive search was performed in accordance with the Preferred Reporting Items for Systematic Reviews and Meta‐Analyses (PRISMA) guidelines. The search was conducted in PubMed (MEDLINE), OVID, EMBASE, the Cochrane Library, Scopus, Web of Science, and Science Direct on 2 August 2019. The date range was restricted to all studies conducted through 2 August 2019. The terms “conditioned media” or “conditioned medium” or secretome or microvesicle or “extracellular vesicle” or exosome and tendon or ligament were used as the search keywords. After removing duplicates and review articles, the titles and abstracts were scanned for eligibility by two authors (T.E.P. and S.R.) independently. Additional searches were performed using the reference lists of the previously included studies. The full text of all selected studies were read by the same authors to apply inclusion and exclusion criteria. Any disagreement between the two authors was resolved by discussion.

### 2.3 Methodological quality assessment and risk of bias

Methodological quality of the included studies was assessed using Animal Research: Reporting of in vivo Experiments (ARRIVE) guidelines.^14^ Hence, we used modified ARRIVE combined with Consolidating Reporting of Trials.^15^ Internal validity was assessed using the Systematic Review Centre for Laboratory Animal Experimentation's risk of bias tool.^16^ Two authors (T. E. P. and S. R.) performed all the assessments independently. Any discrepancy was resolved through discussion with other authors.

### 2.4 Data extraction and synthesis

Two authors (T.E.P. and S.R.) independently recorded data from every included studies. Any disagreements between the two authors were resolved by discussion with other authors. Then, the following data were extracted: study design; type of animal for in vivo studies; establishment process for animals or cells in the included studies; type and specific donor of MSCs; the isolation process for the CM, EVs, or exosomes; interventions; comparison; duration of follow‐up; main outcome for the in vivo studies and the results; any significant differences from control or baseline; and other outcomes.

Any efforts for blinding, if available, would be reported along with the establishment of the animal interventions and follow‐up. For in vivo studies, we reviewed any adverse reactions, quantitative outcome measures analogous to clinical outcome measures, and biomechanical tests as the main outcomes. The authors agreed to classify types of MSCs into BM‐derived MSCs, adipose tissue‐derived MSCs, and amniotic MSCs.

The data collection of in vivo study outcomes are shown in Table 1. Meta‐analysis could not be generated due to the high heterogeneity of the data (ie, source of MSCs, subject animals, outcome measures, and follow‐up duration).

**TABLE 1:** Overview of the studies

Author	Type of MSC (donor)	Level of CM	Animal	Type of controlled laboratory experiments	Animals/cells preparation	Intervention to the main group	Control(s)
Lange‐Consiglio et al^12^	Am‐MSC (horse)	CM	horse	In vivo	13 horses suffering from tendon or ligament injuries were included: 5 were SDFT, 7 SL and 1 DIP	In the range of 8‐30 days postinjury: USG‐guided injection of 2 mL Am‐MSC CM to 10 horses	3 out of the 13 horses were injected with non‐CM solution (not fully stated which were which)
				In vitro	PBMC from centrifugation of horse heparinized whole blood samples. Proliferation was induced by addition of PHA	Addition of 50 and 100 μL/well of Am‐MSC CM	Addition of 50 and 100 μL/well of control
Sevivas et al^1^	BM‐MSC (human)	CM	Rat	In vivo	15 rats underwent bilateral IS and SS tendon resection and left for 16 wk to establish chronic model	Implantation of scaffold combined with human tenocytes + BM‐MSC CM	Scaffold‐only Untreated
				In vitro	Human tendon was obtained from head of the biceps of patients who underwent biceps tenodesis	Tenocytes were incubated with BM‐MSC CM	Incubation with control
Shen et al^13^	Ad‐MSC (mouse)	EV	Mouse	In vivo	32 mice underwent partial transection and subsequent repair of Achilles tendon	Implantation of collagen sheet loaded with EVs from IFN‐γ primed Ad‐MSC (iEVs)	Collagen sheet loaded with EV from naïve Ad‐MSC (nEVs) Collagen sheet only
				In vitro	Macrophages were obtained from BM of femurs and tibiae of adult NGL or FVB mice	Macrophages were pretreated with iEV, nEV, nEV‐free CM, or nEV‐free CM, then treatment with IL‐1β (6 hours, 5 ng/mL)	Pretreatment with control, then treatment with IL‐1β (6 hours, 5 ng/mL)
						Macrophages were pretreated with iEV or nEV, then treatment with IL‐1β (24 hours, 10 ng/mL)	Pretreatment with control, then treatment with IL‐1β (24 hours, 10 ng/mL)
Sun et al^17^	BM‐MSC (human)	CM	Rat (11‐12 w.o.)	In vivo	120 rats underwent ACL resection followed by ACL reconstruction	7 days after injury: CM injection per week	50 μL DMEM injection per week Untreated
				In vitro	NIH3T3 fibroblast was used as a model	Incubation with BM‐MSC CM	Incubation with serum‐free DMEM
Wang et al^11^	TSC (rat)	Exosomes	Rat (8 w.o.)	In vivo	12 rats were injected by type I collagenase solution into both Achilles tendons	Exosomes injection (left side)	PBS injection (right side)
				In vitro	Rat Achilles tendons were dissected and processed to obtain TSC. Identification of TSCs was done using CD34, CD44, CD45, and CD90 immunostaining	Incubation with original TSC CM+IL‐1β or exosome‐free TSC CM+IL‐1β	Incubation with IL‐1β only
						incubation with exosomes+IL‐1β	Incubation with IL‐1β only

Abbreviations: Regarding MSCs: Ad‐MSC, adipose‐derived mesenchymal stem cells; Am‐MSC, amniotic membrane mesenchymal stem cells; BM‐MSC, bone marrow mesenchymal stem cells; TSC, tendon stem cells. Related to preparation procedures: CM, conditioned medium; DMEM, Dulbecco's modified eagle medium; HG‐DMEM, high‐glucose DMEM; EV, extracellular vesicles; MV, microvesicles; FBS, fetal bovine serum. Related to interventions: ACL, anterior cruciate ligament; DIP, distal interphalangeal joint; IS, infraspinatus; SDFT, superficial digital flexor tendon; SL, suspensory ligament; SS, supraspinatus. LPS, lipopolysaccharides.

## 3 RESULTS

### 3.1 Study selection

**FIGURE 1:**
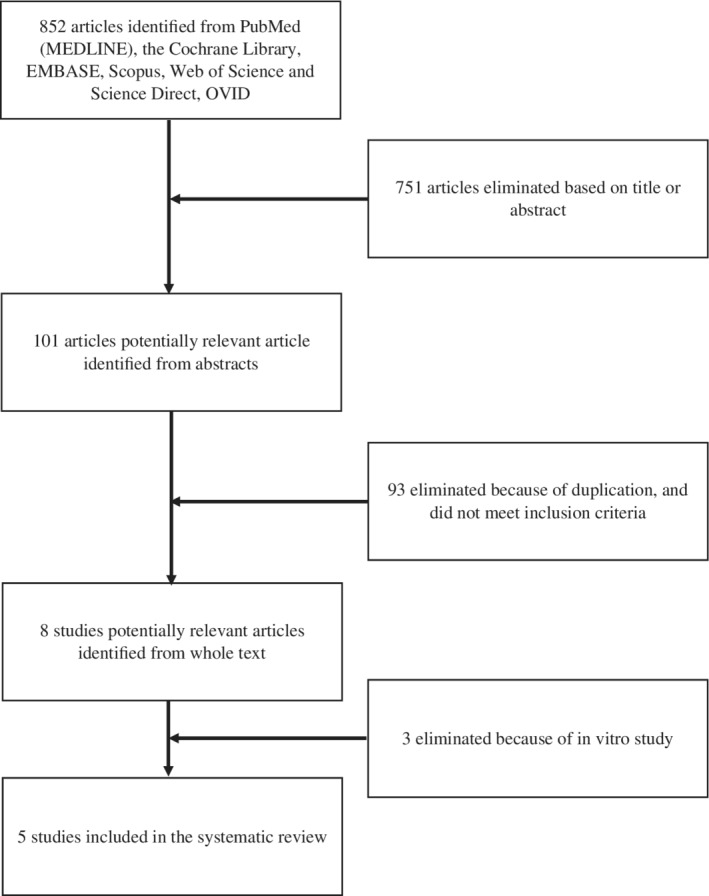
Flowchart of study process selection

A PRISMA flow diagram (Figure 1) summarizes the process of study selection. A total of 852 studies were identified from the literature. After screening of the titles and abstracts, 101 articles were eligible for further evaluation. After full‐text assessment, five studies were included in this systematic review.

### 3.2 Assessment of methodology quality

#### 3.2.1 *Assessment of risk of bias*

##### Study characteristics

An overview of the study is presented in Table 1, and the specific explanation about the study is shown in Table 2, including study design, type of MSC used and their sources, isolation of the CM and its subcomponents, animal injury models for in vivo study, and the interventions.

**TABLE 2:** In vivo studies results

References	Assessment of main outcome	Main outcome measure(s)	Scores/results	Significant difference from baseline	Significant difference between groups	Other evaluation	Other outcome measure(s)
Lange‐Consiglio et al^12^	Monthly	Patient's activity	6 returned to previous activity 3 progressively retrained 2 were included in rehabilitation program 2 were reinjured	N/A	N/A	USG	Lesional echogenity, abnormal tissue evolution
		Adverse reactions to injections	None	N/A	N/A		
Sevivas et al^1^	16 wk	Stiffness (N/mm)	Sc ± CM: 6.25 ± 1.74 Sc only: 6.72 ± 1.28 Untreated: 11.54 ± 2.99	N/A	Sc ± CM<untreated Sc only<untreated Sc ± CM vs Sc only; ND	Histological examination	Ide Modified Tendon Maturing Score
		Total elongation to rupture (mm)	Sc ± CM: 11.99 ± 3.30 Sc only: 9.89 ± 3.47 Untreated: 5.86 ± 3.16	N/A	Sc ± CM > untreated Yes Sc only vs untreated; ND Sc ± CM vs Sc only; ND		
		Adverse reactions to scaffold implantation	None	N/A	N/A		
Shen et al^1^	1 day pretreatment (baseline) 1, 3, and 7 days posttreatment	NF‐κB, 1 day	iEVs: N/S	Yes	No	PCR, histological examination	Proinflammatory genes expression, gap‐rupture rate
			nEVs: N/S	No			
		NF‐κB, 3 days	iEVs: N/S	Yes	No		
			nEVs: N/S	Yes			
		NF‐κB, 7 days	iEVs: N/S	No	Yes		
			nEVs: N/S	Yes			
Sun et al^17^	4 and 8 wk	Stiffness (N/mm), 4 wk	CM: 9.00 ± 1.57. DMEM: 7.09 ± 1.32 Untreated: 7.90 ± 1.11	N/A	CM > DMEM CM vs untreated; ND	MicroCT, histological examination, immunofluorescence imaging	Area of femoral tunnel, area of tibial tunnel, bone volume/total volume ratio, α‐SMA1 area, mean interface width, content of collagen 1
		Stiffness (N/mm), 8 wk	CM: 12.83 ± 2.04 DMEM: 8.51 ± 1.70 Untreated: 8.86 ± 1.15	N/A	CM > DMEM CM > untreated		
		Maximal failure load (N), 4 wk	CM: 5.68 ± 1.13 DMEM: 3.81 ± 0.76 Untreated: 3.49 ± 0.70	N/A	CM > DMEM CM > untreated		
		Maximal failure load (N), 8 wk	CM: 14.91 ± 1.54 DMEM: 10.21 ± 1.12 Untreated: 10.60 ± 1.22	N/A	CM > DMEM CM > untreated		
Wang et al^11^	4 wk	Maximal failure load (N)	N/S	N/A	Exo > Con	Histological examination	Histological score by Stoll et al
		Ultimate stress (N/mm^2^)	N/S	N/A	Exo > Con		
		Breaking elongation (%)	N/S	N/A	Exo vs NI: ND		

Abbreviations: Regarding MSCs: Ad‐MSC, adipose‐derived mesenchymal stem cells; Am‐MSC, amniotic membrane mesenchymal stem cells; BM‐MSC, bone marrow mesenchymal stem cells; TSC, tendon stem cells. Related to isolation procedures: CM, conditioned medium; DMEM, Dulbecco's modified eagle medium; HG‐DMEM, high‐glucose DMEM; EV, extracellular vesicles; MV, microvesicles; FBS, fetal bovine serum. Related to specific tendon/ligament: ACL, anterior cruciate ligament; DIP, distal interphalangeal joint; IS, infraspinatus; SDFT, superficial digital flexor tendon; SL, suspensory ligament; SS, supraspinatus. LPS, lipopolysaccharides.

Most of the studies utilized small animals (three in rats, one in mice). A large animal (horse) was used in only one study. Four studies involved the tendon, and one study involved the anterior cruciate ligament (ACL). Injury models were established by resection, partial resection, collagenase injection, and unintentional tear.

The most commonly used MSC type was from BM (two studies), followed by tendon (one study), adipose tissue (one study), and amnion (one study). Two were from human sources, two from rats, and one from a horse. Conditioned media of the MSCs were isolated using a variety of protocols. The Lange‐Consiglio et al and Shen et al studies separated the EVs from the CM.^12^, ^13^ The Wang et al study obtained the exosomes from the CM.^11^ Shen et al reported that, before isolation of EVs, some MSCs were prepared with IFN‐γ (iEV) and others were not (nEV).^13^

Injections were used to deliver the conditioned media in three in vivo studies.^11^, ^12^, ^17^ The comparison groups were control injection groups (Dulbecco's Modified Eagle's Medium or phosphate buffered saline) and untreated subjects. Wang et al also compared exosomes injection with tendon stem cells (TSCs) injection.^11^ Scaffolds were used to load the conditioned media in two in vivo studies.^1^, ^13^ Sevivas et al compared the treatment with scaffold‐only and untreated groups.^1^ Shen et al compared scaffolds loaded with iEVs vs with nEVs, and with scaffold‐only groups.^13^ Timing of the interventions ranged from immediate to 16 weeks after injury.

##### In vivo study outcomes

In vivo study outcomes are summarized in Table 2, including the main outcomes measures, the scores or results, statements of statistical significance, and a list of other outcome measures.

Among the five studies, functional outcomes were reported in only one study.^12^ In this study, 2 horses that received the nonCM injection were reinjured, whereas 11 horses improved. The differences from the baseline or the control groups were not stated. Regarding safety, Lange‐Consiglio et al reported that there was no adverse reaction after injections of the conditioned media.^12^ Sevivas et al also stated that there was no adverse reaction with scaffold implantations.^1^

Three studies reported biomechanical outcomes. In general, biomechanical outcomes supported the positive effect of conditioned media in tendon and ligament treatment. Sevivas et al reported significant increase in total elongation in tendon rupture treated with scaffold‐loaded CM, compared with a scaffold‐only and an untreated group.^1^ These result was achieved with significant decrease in tendon stiffness. Sun et al found that, 8 weeks after CM treatment, ACL stiffness decreased significantly, whereas the maximal failure load increased significantly compared with both control and untreated groups.^17^ Wang et al reported that in 4 weeks, maximal failure load and ultimate stress were significantly increased in both exosomes and CM injection groups compared with the untreated group^11^; however, there was no difference in breaking elongation in both groups compared with the untreated group.

Other evaluations performed by the authors were imaging studies (ultrasonography and micro CT), histological examinations, immunofluorescence studies, and polymerase chain reaction studies. Despite varied outcome measures, results supported the positive effects of conditioned media in tendon and ligament treatment.

## 4 DISCUSSION

Our primary finding was that MSC CM promoted tendon and ligament healing in preclinical studies. Five in vivo studies showed improvement of functional and biomechanical outcomes in most of the cases. These findings were supported by in vitro studies, in which cell proliferation, viability, and migration were increased in the CM group. Additionally, the differentiation into tenocytes was increased and inflammation was suppressed. Side effects of the application of the CM were reported in only two studies, but the side effect itself was not stated. Moreover, no study directly compared a CM group to an MSC group.

This study showed that CM was almost always associated with significantly better functional and biomechanical outcomes than the control groups. Shen et al, Sun et al, and Wang et al were in agreement that CM resulted in better biomechanical outcomes.^11^, ^13^, ^17^ A systematic review by Ahmad et al^4^ also found biomechanical benefit using various MSCs for tendon repair and regeneration. Indeed, every type of MSC has shown regenerative potential and given phenotype characteristics. CM composition varied depending on the type of cultured MSC.^5^, ^18^ Therapeutic effects of MSCs are largely attributed to paracrine pathways. The EVs, exosomes, and even its microRNAs, the subcomponent of CM, have also been found to contribute to their therapeutic and anti‐inflammatory effects, but a detailed mechanism of action needs to be elucidated.^6^ Scaffolds alone may be beneficial for cell health and attachment because of its structural approximation of the original tissue.^19^, ^20^ These effects are further enhanced by the addition of MSCs or its CM.^13^, ^21^, ^22^

The included in vitro studies support the positive effect of CM in tendon and ligament healing. Two studies showed significantly higher tenocyte proliferation, whereas two other studies showed significant increase in cell viability. A study by Shimode et al found that the regenerative benefit was obtained from the paracrine effect of the MSC only, not necessarily because of the CM derived from the MSCs and tenocytes coculture.^10^ Inflammation markers were found to be suppressed in three studies.^11^, ^12^, ^13^ Additionally, Shen et al and Wang et al found that inflammation markers are significantly higher in exosome or EV‐deprived CM than in unprocessed CM and that these deprived CM were no different than the control.^11^, ^13^ Interestingly, Lange‐Consiglio et al found that there was no difference in PBMC proliferation in groups treated with EV and the control group in vitro^12^; however, in their study, the CM and supernatants of the CM decreased PBMC proliferation significantly, which suggests immunosuppressing effects of the soluble factors.^12^ In fact, EV effects on immunomodulation varies across studies, with some studies finding that there were no differences compared with control and other studies reporting comparable results to the CM.^23^, ^24^, ^25^, ^26^ Thus, general consensus has not been reached and further high‐quality research is needed to confirm EV functions.

There were several limitations to this study. First, among the five included studies, only one study reported functional outcome with nonrandomized subjects. Second, these studies utilized different cell sources, level of conditioned media (CM, EV, or exosome), and delivery methods. Different types of animals were used for the in vivo studies. This heterogeneity clearly will cause different outcomes. The studies used in our review varied considerably in terms of MSC sources (adipose, BM, and TSCs), different level of conditioned media (the pure CM, exosome, and EV), and different delivery methods (with or without scaffold implantation). Finally, the assessment tools also widely varied between studies. Therefore, it was impossible to perform a quantitative analysis with the included studies. Further research is needed to understand how CM and its subcomponent play a role in tendon and ligament healing.

## 5 CONCLUSION

Utilization of stem cells CM (secretome) was promising in enhancing the tendon and ligament healing process, as shown by the available preclinical studies; however, more preclinical studies are needed to further confirm the benefits and to produce the most suitable CM for future clinical studies.

## CONFLICT OF INTEREST

The authors declared no potential conflicts of interest.

## AUTHOR CONTRIBUTIONS

S.R.: research concept, literature search, data analysis, manuscript preparation, drafting the manuscript, reviewing and editing the manuscript; T.E.P.: research concept, literature search, data analysis, manuscript preparation, drafting the manuscript, reviewing and editing the manuscript, visualizing the data into table; R.S.: research concept, data analysis, manuscript preparation, drafting the manuscript, reviewing and editing the manuscript, visualizing the data into table; N.R.S., C.R.S.P.: data analysis, manuscript preparation, drafting the manuscript, reviewing and editing the manuscript; F.N.U.R., A.P.S., T.S., D.N.U., H.S., M.F.: data analysis, manuscript preparation, drafting the manuscript.

## Data Availability

Data sharing is not applicable to this article as no new data were created or analyzed in this study.
